# Personalized Dosimetry in Targeted Radiation Therapy: A Look to Methods, Tools and Critical Aspects

**DOI:** 10.3390/jpm12020205

**Published:** 2022-02-02

**Authors:** Rachele Danieli, Alessia Milano, Salvatore Gallo, Ivan Veronese, Alessandro Lascialfari, Luca Indovina, Francesca Botta, Mahila Ferrari, Alessandro Cicchetti, Davide Raspanti, Marta Cremonesi

**Affiliations:** 1 Dipartimento di Fisica, Università degli Studi di Pavia, Via Bassi 6, 27100 Pavia, Italy; rachele.danieli01@universitadipavia.it; 2 Fondazione Policlinico Universitario A. Gemelli IRCCS, Largo F. Vito 1, 00168 Roma, Italy; luca.indovina@policlinicogemelli.it; 3 Dipartimento di Diagnostica per Immagini, Radioterapia Oncologica ed Ematologia, Università Cattolica del Sacro Cuore, Largo F. Vito 1, 00168 Roma, Italy; 4 Dipartimento di Fisica “Aldo Pontremoli”, Università degli Studi di Milano, Via Celoria 16, 20133 Milano, Italy; salvatore.gallo@unimi.it (S.G.); ivan.veronese@unimi.it (I.V.); 5 INFN Sezione di Milano, Via Celoria 16, 20133 Milano, Italy; 6 INFN-Pavia Unit, Department of Physics, University of Pavia, Via Bassi 6, 27100 Pavia, Italy; alessandro.lascialfari@unipv.it; 7 Medical Physics Unit, European Institute of Oncology IRCCS, Via Giuseppe Ripamonti 435, 20141 Milano, Italy; francesca.botta@ieo.it (F.B.); mahila.ferrari@ieo.it (M.F.); 8 Prostate Cancer Program, Fondazione IRCCS Istituto Nazionale dei Tumori, Via Giacomo Venezian, 1, 20133 Milano, Italy; Alessandro.Cicchetti@istitutotumori.mi.it; 9 Temasinergie S.p.A., Via Marcello Malpighi 120, 48018 Faenza, Italy; davide.raspanti@temasinergie.com; 10 Radiation Research Unit, European Institute of Oncology IRCCS, Via Giuseppe Ripamonti 435, 20141 Milano, Italy; marta.cremonesi@ieo.it

**Keywords:** targeted radiation therapy, molecular radiation therapy, selective intra-arterial radiation therapy, treatment planning systems, personalized dosimetry

## Abstract

Targeted radiation therapy (TRT) is a strategy increasingly adopted for the treatment of different types of cancer. The urge for optimization, as stated by the European Council Directive (2013/59/EURATOM), requires the implementation of a personalized dosimetric approach, similar to what already happens in external beam radiation therapy (EBRT). The purpose of this paper is to provide a thorough introduction to the field of personalized dosimetry in TRT, explaining its rationale in the context of optimization and describing the currently available methodologies. After listing the main therapies currently employed, the clinical workflow for the absorbed dose calculation is described, based on works of the most experienced authors in the literature and recent guidelines. Moreover, the widespread software packages for internal dosimetry are presented and critical aspects discussed. Overall, a selection of the most important and recent articles about this topic is provided.

## 1. Introduction

Targeted radiation therapy (TRT) is one of the available options for the treatment of benign, malignant or inflammatory diseases [1]. TRT is based on the administration of a radiopharmaceutical, i.e., an agent composed of an α/β-emitting radionuclide usually bound to an active or inert vector molecule. Radiopharmaceuticals are mostly injected intravenously or through intra-arterial or oral access. TRTs can be divided into two subgroups, depending on how the radiopharmaceutical reaches the tumoral site: molecular radiation therapy (MRT) [2] and selective intra-arterial radiation therapy (SIRT) [3]. The former uses the natural tropism of a radionuclide (e.g., ^131^I for the thyroid or ^223^Ra for bone tissue) or a vector molecule (e.g., PSMA or DOTATATE) selected according to biochemical properties of the disease (e.g., overexpression of specific receptors) so that the radiopharmaceutical binds preferentially to the target cells. The latter, instead, exploits the tumor vascularization: the radiopharmaceutical is directly injected, in the form of microspheres, into the tumor arterial blood circle and no active molecule is needed. The damage to the targeted cells is obtained through the emitted radiation and is expressed in terms of a physical quantity called absorbed dose, which is defined as the radiation energy absorbed per unit mass.

At present, especially in MRT, most hospital centers administer a fixed or weight-scaled amount of activity, regardless of the absorbed dose to the volumes of interest (VOIs) [4]. However, the same administered activity corresponds to different absorbed doses in normal organs and target tissues of different patients and also among various lesions of the same patient [5,6]. Moreover, since there is increasing evidence that the treatment outcome—both in terms of efficacy and toxicity—is related to the radiation released, i.e., the absorbed dose rather than to the administered activity [7], the fixed-activity approach can easily lead to undertreatment or overtreatment of patients. This plays against the principle of optimization required by the European Council Directive (2013/59/EURATOM) [8], according to which TRT treatment must be optimized for each patient, similar to what is already used in external beam radiation therapy (EBRT). 

Dosimetry is particularly important for therapies aimed to treat cancer, which are rapidly developing and for which the risk–benefit ratio has still to be carefully evaluated. As a consequence, this paper focuses on those applications, ignoring therapies employed for the treatment of benign or inflammatory diseases.

In the context of TRT, a treatment planning consists in prescribing the activity to administer based on threshold doses to organs at risk (OARs) and, if possible, to lesions [4]. The implementation of personalized dosimetric protocols is thus fundamental. Currently, threshold doses are extrapolated from EBRT literature for which normal tissue complication probability (NTCP) curves are available. However, a proper optimization requires the development of radiobiological models specific for each TRT, since there is increasing evidence suggesting that the mechanisms of cellular response to low vs. high dose-rate exposures are different [9,10,11]. 

After the treatment, post-therapy dosimetry should also be performed in order to verify the delivered absorbed dose. 

The European regulation directly impacts the work of medical physicists and nuclear medicine physicians, and the request for educational resources regarding internal dosimetry is particularly high. Arising from this need, the present paper focuses on the available methods and tools for personalized dosimetry in TRT and is addressed to physicists, medical physicists and everyone working in or approaching this field, with the aim to provide a general overview of this rapidly evolving and active discipline. 

## 2. Radiopharmaceuticals in TRT

Different radiopharmaceuticals can be used in TRT, according to the treated disease [12]. Those most applied are summarized in Table 1. 

The calculation of the absorbed dose requires the assessment of the activity distribution throughout the patient body over time. In some cases, the radionuclide used for the therapy presents itself an emission channel (e.g., γ, e^+^) or a paramagnetic component that can be used for tracking the substance with the current imaging systems (planar scintigraphy, SPECT, PET, MRI). In this scenario, if the treatment consists of a single administration (e.g., ^131^I for thyroid diseases, ^131^I-MoAbs for lymphomas), dosimetry is usually performed before therapy administering a tracer activity, i.e., a low amount of the radiopharmaceutical itself, focusing attention on not altering the target uptake of the subsequent therapy, i.e., avoiding the so-called “stunning effect” [13,14]. Furthermore, the difference in the setup sensitivity when moving from diagnostic images at low activity to treatment images at high activity could in principle introduce errors in the assessment of the absorbed dose [15]. 

If multiple cycles are planned, and if the radionuclide is suitable, dosimetry can be performed during the therapy, at the first and/or subsequent cycles (e.g., ^177^Lu-DOTATATE for neuroendocrine tumors, ^177^Lu-PSMA for prostate cancer). Dosimetry performed only after a single cycle is usually associated with the hypothesis that the radiopharmaceutical biodistribution in subsequent cycles remains unaltered, but this approximation could introduce errors in the evaluation of the absorbed dose [15,16,17]. 

If the radionuclide is not suitable for dosimetry (i.e., presenting a lack or very low abundance of gamma or positron emissions), instead, other radionuclides with a physical half-life (T_1/2_) compatible with the biological half-life of the vector and similar chemical properties represent an important option for provisional dosimetry in MRT (e.g., ^111^In-DOTATOC/^90^Y-DOTATOC for neuroendocrine tumors). In this scenario, the inability of the surrogate radiopharmaceutical to reproduce the exact pharmacokinetics of the therapeutic compound must be carefully evaluated [18]. 

In SIRT, since the effective half-life of the surrogate radiopharmaceutical is equal to the physical one, a proper distribution mimicking requires that the emitting compound comes in similar size and number of the therapeutic microspheres (e.g., ^99m^Tc-MAA for ^90^Y radioembolization). In this particular case, furthermore, the high activity concentration of ^90^Y in the tumoral regions during the treatment also allows performance of a post-therapy dosimetry with ^90^Y-PET images, despite the extremely low positron channel branching ratio. The comparison of provisional and post-therapy dosimetry remains a matter of investigation [19,20], and strongly depends on the intra-arterial procedure and repeatability. 

The lack or very low abundance of gamma emission represents a typical drawback of alpha emitter therapies such as ^223^Ra, ^225^Ac and ^213^Bi-MoAbs and ^225^Ac-PSMA, jeopardizing the feasibility or reliability of individual dosimetry. In order to overcome this problem, some authors have proposed extrapolations from the dosimetry using the same molecule and different radionuclides, i.e., assuming similar uptake and retention [21,22]. As a general rule, the reliability of the dosimetry information derived must always be assessed specifically for each type of radiolabeled molecule, since different radionuclides might influence the radiochemical stability and the biokinetics. This issue can be addressed in preclinical studies.

## 3. One Activity Does Not Fit All

A one-size-fits-all approach based on the administration of a fixed or weight-scaled amount of activity is usually adopted in the field of radiation therapy from internal sources. Clinical trials based on escalating administered activities are used to collect data on possible toxicity (especially early) and efficacy, from which define standard activity amounts to administer (e.g., SIRT with ^90^Y-microspheres, ^177^Lu-DOTATATE). However, different studies demonstrated that the same administered activity corresponds to a wide range of absorbed doses to target and nontarget volumes, caused by the interpatient variation of metabolism (Table 2). In addition, since for an increasing number of therapies there is reason to believe that the absorbed dose is related to the treatment outcome (Table 3), the wide range of absorbed doses per injected activity suggests a risk of under dosage of tumoral lesions and an overexposure to normal tissues for a portion of patients and that the potential of the radiopharmaceutical is not fully exploited. 

The choice of using one-size-fits-all protocols was comprehensible in the early years of TRT, considering the simplicity of the method and the lack of studies regarding internal dosimetry, but currently it is highly questionable. Although a number of questions have to be further investigated, most results in literature suggest that the one-size-fits-all approach appears inadequate and does not guarantee either optimization or best standard of care for the patient [33].

## 4. How to Calculate the Absorbed Dose

The calculation of the absorbed dose to normal organs and target regions requires two major ingredients: 

(i) The activity distribution inside the patient body over time to assess, through time integration, the total number of disintegrations occurring in each region of interest (cumulated activity);

(ii) The computation of conversion factors, or direct MC simulations, for changing cumulated activity into absorbed dose. 

**Figure 1:**
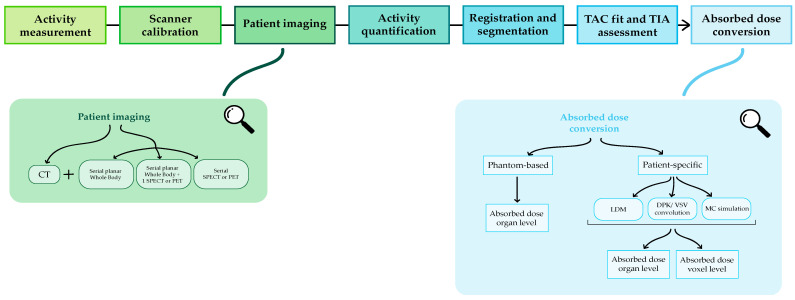
MRT clinical dosimetric workflow.

Therefore, this calculation should not be thought of as an isolated process but as part of a dosimetric workflow, the main steps of which are represented in Figure 1.

SIRT represents a particularly simple context for dose calculation. In fact, the microspheres reach the tumoral site exploiting liver vascularization and they remain trapped in the lesion vessels; therefore, metabolic kinetic is not present and imaging at multiple timepoints is not needed. As a consequence, some steps of the dosimetric workflow described in Figure 1 are not required for the dose calculation.

### 4.1. Activity Measurement

As preliminary operation, the activity inside the vials for pre-therapeutic imaging or treatment have to be measured with a dose calibrator [51]. An accurate analysis of its sensitivity in response to different geometries is suggested, along with a proper calibration for each radionuclide of interest. 

The activity measurement with the dose calibrator allows not only assessment of the administered activity analyzing the vial residual, but also performance verification of the nominal value provided by the supplier and to measure the activity inside phantoms for scanner calibration or image quantification. 

### 4.2. Scanner Calibration

The first step in the dosimetric workflow is the determination of a calibration factor for the scanner, i.e., a factor for the conversion of the count rate (cps) into absolute activity (MBq). Different methods for the assessment of this factor have been proposed and are currently used [52]. The standard calibration procedure consists of preparing a radioactive sample of well-determined activity (measured with the dose calibrator) and detecting its count rate. The ratio between the count rate and the known activity gives the calibration factor. 

The calibration factor obviously depends on the considered radionuclide, on the size and shape of the sample (point-source or active phantom) and on the imaging technique employed (planar scintigraphy or tomography). Furthermore, it is good practice to assess it periodically in order to detect any possible variation. 

### 4.3. Patient Image Acquisition

According to the endpoint, type of treatment and institute machine availability, different imaging protocols can be used in order to assess the activity distribution throughout the patient body: planar, hybrid and 3D protocol [53]. 

Planar protocol requires the acquisition of sequential whole-body 2D images. This approach does not allow for a reliable activity determination in overlapping structures and it enables only the calculation of the mean absorbed dose to organs or lesions. As advantages, it is very fast and easy to implement. Furthermore, it easily provides whole-body images, which are fundamental, e.g., in the presence of diffuse metastasis.

In order to overcome the limitations of the planar protocol and to determine the absorbed dose at the voxel level, i.e., to obtain dose maps, one additional SPECT/CT or PET/CT can be acquired at one of the timepoints to quantify the activity and then combine it with the activity variation vs. time derived from serial planar images (hybrid protocol) [54,55]. As a third option, multiple SPECT/CTs or PET/CTs can be used (3D protocol) for complete 3D information, although more than one bed per time might be required due to the limited field of view. 

### 4.4. Activity Quantification

Once the images have been acquired according to one of the protocols noted above, the absolute activity inside each of the source regions, i.e., regions in which the activity cumulates significantly, has to be determined. In the case of SPECT or PET images, raw data acquired in projections have to be reconstructed. Different iterative algorithms have been developed and are currently used in clinical routine for this purpose [56]. Furthermore, since the imaging procedure inevitably introduces errors both in terms of loss and displacement of the signal, a set of corrections should be applied to the images in order to recover the true counts. Those corrections include attenuation, scatter, dead time, collimator-detector response and partial volume effect. Different methods are available for image correction, either for planar and tomographic images [57,58,59]. The same kind of image corrections should also be applied during the scanner calibration procedure.

### 4.5. Registration and Segmentation

Volume of interest (VOI) or region of interest (ROI) must be defined on a reference scan using manual, semiautomatic or automatic tools. Then, images at different timepoints are registered to the reference scan using rigid (translation and rotation) and/or elastic algorithms and segmentations are propagated. As an alternative, segmentation can be performed for each of the timepoint images. 

For SPECT/CT or PET/CT acquisition, the registration algorithm is usually applied to the CT scan series and then merged/fused with SPECT or PET image, with the CT less affected by a possible error due to the higher spatial resolution. Although different and increasingly advanced methods have been developed, registration is still challenging, especially in case of organ motion, variation in shape and size.

In SIRT, since in principle the kinetic process is not involved, imaging at multiple timepoints is not needed (only physical half-life applies) and registration refers only to multiple imaging modalities.

### 4.6. Time Activity Curve (TAC) Fit and Time Integrated Activity (TIA) Assessment

After segmentation and registration, the activity in source regions at different timepoints is known and the cumulated activity ${\tilde{A}}_{r_{S}}$ inside the source regions can be determined. Analytical methods or linear interpolation (trapezoidal method) are commonly used for approximating the time–activity curve (TAC) between the first and last experimental timepoints. In the first case, sums of exponentials are often used as mathematical functions for fitting the time–activity curves $A_{r_{S}}\left(t\right)$ [60]:

(1) $$A_{r_{S}}\left(t\right)=\underset{j}{\sum }A_{j}\left(0\right)e^{\begin{array}{c} -\left(\lambda +\lambda_{j}\right)t \end{array}}$$

where $A_{j}\left(0\right)$ is the initial activity value of the jth exponential component, λ is the physical decay constant related to the physical half-life $T_{1/2}$ of the radionuclide through the relationship $\lambda =0.693/T_{1/2}$ and $\lambda_{j}$ is the biologic elimination constant corresponding to the biologic half-life $T_{1/2,j}$ ($\lambda_{j}=0.693/T_{1/2,j}$) of the jth exponential component; $\left(\lambda +\lambda_{j}\right)$ represents the effective elimination constant.

**Figure 2:**
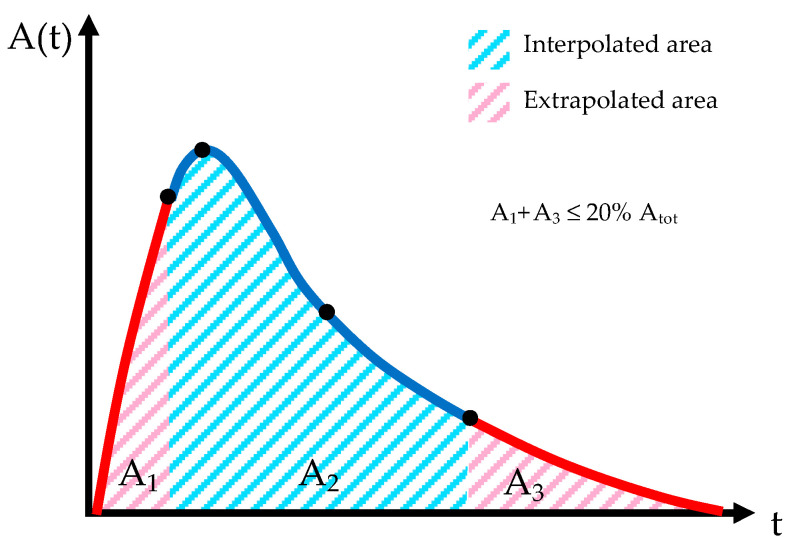
Time–activity curve (TAC) with interpolation and extrapolation areas.

Constant, linear, or analytical fit are usually proposed for the extrapolation of the time–activity curve before the first experimental timepoint, whereas analytical or physical decay are options for extrapolating the curve after the last timepoint (Figure 2). Appropriate extrapolation of the curve in order to obtain the time-integrated activity (TIA) is crucial for dosimetry accuracy [61]. In order to avoid possible significant errors, therefore, the European Association of Nuclear Medicine (EANM) guidelines recommend that the fractional contribution of the TIA from the extrapolations should be less than 20% [62]. 

Integration of the TAC could be performed both at the organ, i.e., considering the mean activity inside a macroscopic source region, and at the voxel level, i.e., considering the mean activity inside a single voxel. In the first case, a single pharmacokinetics behavior, i.e., unique TAC parameters, is assessed for the whole region; in the second case, instead, different behaviors are determined for each voxel.

As for registration between different timepoints, TAC fit and integration does not refer to SIRT.

### 4.7. Absorbed Dose Conversion

Different methods for converting the cumulated activities ${\tilde{A}}_{r_{S}}$ into absorbed dose are available. 

The MIRD formalism, developed by the Medical Internal Radiation Dose (MIRD) Committee of the Society of Nuclear Medicine (SNM), was the first dosimetric tool and it allowed the assessment of the mean absorbed doses in organs and tumors [63]. The fundamental equation of the MIRD approach follows:

(2) $$D\left(r_{T}\right)=\overset{}{\underset{r_{S}}{\sum }}\tilde{A}\left(r_{S}\right)\cdot S\left(r_{T}\leftarrow r_{S}\right)$$

where $D\left(r_{T}\right)$ is the absorbed dose delivered to the target region $r_{T}$, $\tilde{A}\left(r_{S}\right)$ is the time-integrated activity (TIA) in the source region $r_{S}$ and $S\left(r_{T}\leftarrow r_{S}\right)$ is the mean absorbed dose to $r_{T}$ per unit activity present in $r_{S}$, called S-value. 

The TIAs reflect the patient-specific biodistribution of the considered radiopharmaceutical while the S-values are instead based exclusively on the physical features of the radionuclide selected for the treatment and on the characteristics of the target and source regions. They can be expressed as where $E_{i}$ is the emitted energy (mean or individual) for the *i*th nuclear transition, $Y_{i}$ is the probability of the ith nuclear transition, $\phi_{i}\left(r_{T}\leftarrow r_{S}\right)$ is the fraction of $E_{i}$ emitted within the source tissue $r_{S}$ that is absorbed in the target tissue $r_{T}$ and $m_{r_{T}}$ is the mass of the target region:

(3) $$S\left(r_{T}\leftarrow r_{S}\right)=\frac{\sum_{i}E_{i}Y_{i}\phi_{i}\left(r_{T}\leftarrow r_{S}\right)}{m_{r_{T}}}$$

S-values for different radionuclides and source-target combinations have been calculated using MC codes and reference anthropomorphic computational phantoms with homogeneous density for each tissue and uniform activity inside each region. For tumors, a spherical model is provided with S-values for spherical regions of various volumes. However, as general phantoms cannot model the many diverse scenarios (i.e., number of tumors, site and volume), any cross-fire contributions arising from regions other than the tumor itself are set to zero.

Despite its strict assumptions of tissue homogeneity and uniform activity distribution, the MIRD approach is still diffused worldwide, due to its fast calculations and the possibility to use planar images in addition to SPECT/PET. In order to overcome its limitations, however, methods which consider the patient-specific activity distribution derived from functional images (SPECT or PET) have been developed [64]. Those methods provide, in addition to mean doses to target organs and lesion, dose maps and dose–volume histograms (DVHs).

**Figure 3:**
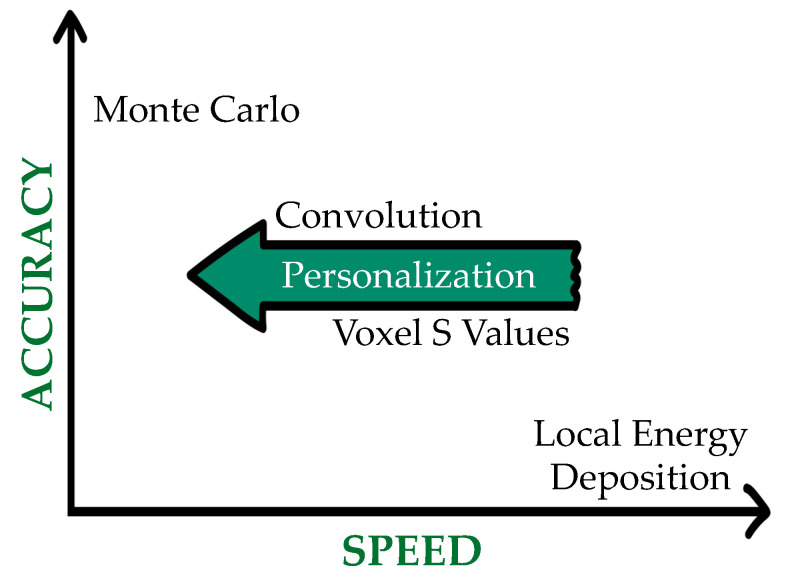
Dosimetric approaches in a figurative accuracy–speed plane.

The most accurate technique for absorbed dose assessment, at least theoretically [65], is a direct Monte Carlo simulation of radiation transport, which can account for both nonuniform activity and heterogeneous tissues (Figure 3). In this approach, the activity map is used to sample the decay location and the transport of the radiation emitted from those sites into the patient-specific geometry derived from the CT, which straightly allows for the calculation of the energy deposited in each voxel. MC simulations require numerous parameters and the computation time can be intensive, depending on the number of primaries selected for the simulation and on the hardware specifics. The MC codes most used for internal dosimetry are GATE/Geant4 [66], EGSnrc [67], MCNPX [68,69] and Fluka [70].

After MC, convolution with dose-point kernel (DPK) or voxel S-values (VSV) is in principle the most accurate method for dose calculation at the voxel level. A DPK represents the mean absorbed dose per transition (mGy/MBq/s) at a given radial distance from an isotropic point source located within a homogeneous, infinite medium (typically water). DPKs are continuous functions and in order to be used for voxel dosimetry they must be discretized and adapted to the voxel geometry. This could be done either considering source and target voxels as collapsed to the voxel centroid or as entire volumes. In this last case, a multidimensional integration of DPKs over the source and target voxels must be performed. The convolution with the cumulated activity map results in a dose map. 

The VSV approach was introduced by the MIRD Pamphlet No. 17 and is the analogue at the voxel level of the MIRD formalism at the organ level. In fact, nothing in principle prevents the MIRD schema to be applied to smaller volumes, i.e., sub-organs or even cells, except for the fact that the resolution of the PET or SPECT images must be adequate. The main equation is therefore a generalization of (2):

(4) $$D\left(voxel_{k}\right)=\overset{N}{\underset{h=0_{}}{\sum }}\tilde{A}\left(voxel_{h}\right)\cdot S\left(voxel_{k}\leftarrow voxel_{h}\right)$$

where $voxel_{k}$ is the target voxel and $voxel_{h}$ is one of the *N* source voxels.

VSV are calculated with a direct MC simulation of radiation transport into a homogeneous, infinite medium and into voxelized geometry. The convolution of those factors with the activity map gives the 3D dose distribution.

VSV have been calculated for different voxel sizes, radionuclides and medium [64,71]. For the most used radionuclides, VSV kernels are usually sized 7 × 7 × 7 or 11 × 11 × 11. In order to overcome the limitations of VSV tabulations due to the voxel size and shape, other methods have been developed, such as the fine resolution and resampling method developed by Dieudonné et al. [72], the analytical model by Amato [73] or the DPK integration [74].

The assumption of homogeneous tissue compositions in DPK and VSV calculation may lead to substantial errors in dose distributions when regions of the body with high tissue heterogeneities are considered (i.e., air–tissue or bone–tissue interfaces). In those cases, direct MC simulations can be used.

Finally, the local energy deposition method (LDM) assumes that all the energy is absorbed in the source voxel. As a consequence, the LDM can be used when the voxel dimension is greater than the radiation range, typically when alpha and short-range beta emitters are considered. For photons or “high” energy beta emitters with range larger than voxel dimensions, the convolution of VSV or a direct MC simulation should be more adequate, at least theoretically. However, due to the limited resolution of SPECT and PET images, the LDM might be preferable in some cases, as the former is considered to cause a further blurring of the images and for the latter the longer computation time and complexity might not be justified [65,75,76]. 

### 4.8. Dose-Rate Integration

**Figure 4:**
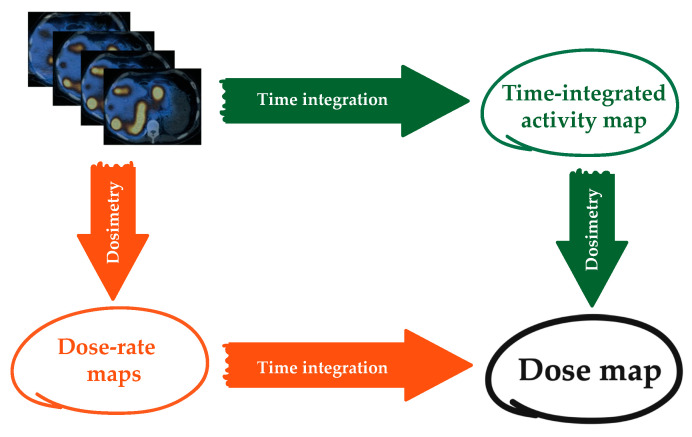
Concept map of the two possible approaches to calculate an absorbed dose map.

As an alternative to the activity integration, time integration of the dose rate could be also performed. In this case, activity maps at each time point are converted into dose-rate maps using one of the methods described above and absorbed dose is obtained by integrating the dose rate (Figure 4). 

Although there are not yet studies investigating this point, it seems that absorbed dose values obtained by time-integrating the absorbed dose rate are more reliable than those obtained through the integration of the TAC. One possible reason could be that the dose rate can be thought of as a smoother function, being normalized for the mass, and thus easier to integrate. In addition, the activity integration is performed assuming that the VOIs in which the activity is evaluated at each timepoint are the same, but patient movement, organ deformation and the low resolution of the images make it impossible. On the contrary, being that the dose rate is already divided by the mass, it becomes independent of the possible variation of the VOI volumes in the serial images. Finally, it must be considered that in therapy the absorbed dose rate could be probably a more significant dosimetric quantity as compared to the absorbed dose to be associated with the radiation effect, from a radiobiological perspective. As a consequence, it seems more reasonable to perform the calculation of the absorbed dose rate instead of that of the cumulated activities. This issue is presently under investigation.

### 4.9. Uncertainties

Each step of the dosimetric workflow described above is associated with a specific uncertainty (e.g., uncertainty in the volume determination, number of counts, calibration factor, fitting parameters and S factors), all of which are combined in a complex manner to determine the global uncertainty related to the absorbed dose itself. The EANM provided guidelines for calculating the absorbed dose uncertainty based on the law of propagation of uncertainties [77]. Following those indications, Finocchiaro et al., e.g., estimated the uncertainty associated with the tumor absorbed dose in patient treated with ^177^Lu-DOTATATE, which exceeded 100% [78].

Although an uncertainty analysis is not currently included in the clinical routine, its practice would help to identify and reduce errors, and make data collected in different centers comparable [79].

## 5. The Potential Role of Artificial Intelligence in TRT

Artificial intelligence (AI) is increasingly applied in medical physics [80], including internal dosimetry [81,82]. For TRT, the most significant application of AI consists in the development of fully automated segmentation tools [83,84,85], which contribute not only to reduce the overall time required for the dosimetry evaluation, but also to reduce the user-dependent variability associated with the absorbed dose. 

The involvement of AI into the other steps of the dosimetric workflow (e.g., image acquisition, reconstruction, registration and absorbed dose conversion) is very challenging and currently under investigation. Some authors used AI to develop density specific S-values able to overcome the limitation of standard VSV calculated into homogeneous mediums with the aim to enhance the dose calculation accuracy [86,87]. Other groups studied the accuracy of AI methods to convert the activity images directly into dose-rate maps [88,89]. Finally, the possibility of predicting the absorbed dose starting from diagnostic images was also investigated [90]. The validation of this method, however, would require a consistent number of training datasets for different therapies and clinical situations, still not available or adequately collected. The assessment of robust and more standardized dosimetry methods, which is a priority and matter of most present efforts by the internal dosimetry community, will certainly open the way to the implementation of IA in TRT dosimetry.

## 6. Software Packages for Internal Dosimetry

Many software packages for internal dosimetry have been developed throughout the last years, either homemade or commercially available. Most are meant for research purposes only, but some detain the Food and Drug Administration (FDA) approval and/or the *conformité européenne* (CE) marking and are intended for clinical use. Different packages may address different parts of the dosimetric workflow. Some only provide tools for converting TIA into absorbed dose, others include registration and segmentation and others also allow for quantifying the activity, including algorithms for image reconstruction and correction.

Software packages can be basically divided into two groups: a first-generation which perform dosimetry at the organ level, i.e., providing only mean doses to organs and lesions, and more sophisticated and recent packages that perform dosimetry at the voxel level, which also provide a dose map and DVH. In the first group, the activity distribution inside the source regions is supposed to be uniform, whereas the programs of the second group use the patient activity distribution in SPECT or PET images to derive the dose map.

### 6.1. Organ Level or Phantom-Based Software Packages

Software packages at the organ level use the traditional MIRD scheme and assume homogeneous tissue density and composition and a uniform activity distribution inside each source organ or region of interest. Libraries with specific absorbed fractions (SAFs) and the S-values previously calculated with MC codes for various anthropomorphic phantoms [91,92,93] are loaded into the software, along with radionuclide decay data [94,95]. The user needs to select the radionuclide and the phantoms of interest and to provide the time-integrated activity coefficients (TIACs), i.e., the TIA divided by the administered activity, for each of the source organs. Output data are the average absorbed doses and/or the equivalent doses per unit administered activity for each of the target organs. 

Anthropomorphic phantoms incorporate reference data [96] reproducing the average characteristics of a population. Most of the available software, however, include the possibility to take a first step into personalization correcting the absorbed dose for the patient-specific organ masses [97]. A further step in this direction is provided by MIRDcalc, which employs weight-based phantoms accounting for different geometries. In such phantoms the organ masses and S-values are linearly scaled from the two reference phantoms (ICRP phantoms) closest by mass to the patient (https://mirdsoft.org/mirdcalc, accessed on 12 December 2021) [98,99]. 

Since tumors are not included in anthropomorphic phantoms, S-factors for isolated (i.e., considering only self-dose) unit density spheres of different volumes have been calculated and loaded into most of the software packages.

In Table 4 the most widely used software packages for dosimetry at the organ level are summarized, along with their main characteristics.

Among the software packages mentioned above, OLINDA/EXM [102,103] and 3D-RD-S (http://rapiddosimetry.com, accessed on 12 December 2021) are the ones providing a tool to perform kinetic data analysis. OLINDA/EXM allows the user to enter kinetic data and fits them with a multiexponential model to derive the integral activity. Parameters about the goodness of the fit are not provided, and the accuracy of the residence time estimation is left to user expertise. 3D-RD-S was recently developed and offers different options for fitting and integrating the TAC, providing parameters about the goodness of the fit and uncertainties. 

A new free software at the organ level is being developed by the OPENDOSE collaboration [104], meant for diagnostic applications and radiation protection and presently under validation. As a novelty, it allows the user to choose among different radiation spectra, different phantoms (with associated radiation protection parameters) and different SAF values, including those provided by OPENDOSE itself with uncertainty estimates [105]. 

Software packages at the organ level have been a most important tool for internal dosimetry in the last 30 years, allowing its diffusion and daily practice within medical centers for their low cost, user-friendliness, extremely short calculation time and no special computer requisites. However, since uniform activity distribution within source organs is assumed, patient-specific dosimetric approaches, i.e., approaches in which the activity distribution derived directly from SPECT or PET images is maintained, are currently required in order to perform a more reliable personalized dosimetry.

### 6.2. Voxel Level or Patient-Based Software Packages

Different software packages for the voxel level dosimetry (3D or hybrid) have been recently developed, both for MRT and SIRT. Although they are usually classified on the basis of the method used to create the dose map, software packages for dosimetry typically differ not only in this but also in the tools they offer for registration, segmentation and integration of the TACs. Comparisons among different software packages are thus needed to point out these differences and possibly reduce them in view of a standardization of the dosimetric procedure [106,107,108].

The main commercial software packages for 3D dosimetry are reported in Table 5.

## 7. Discussion

The implementation of protocols for patient-specific dosimetry—intended as both treatment planning (pre-therapy) and verification (post-therapy) dosimetry—requires economic investments from hospitals (e.g., money for advanced and adequate technical devices, software packages and qualified personnel), educational efforts and patient compliance for further analysis and investigations [15,109]. This practice, thus, should prove to be advantageous in terms of costs and benefits in order to be introduced into the clinical routine.

According to a recent European survey, the implementation of dosimetry-based treatment planning is poorly diffused [109]. In most cases, the absence of dose-effect correlations—and thus of dose thresholds for OAR toxicity or tumor response—seems to justify the standard practice based on fixed or weighted-based activity. However, the lack of dose-effect evidence could be in some cases only illusory. Unsuitable dosimetric methods (e.g., related to calibration, image corrections, fitting, use of surrogate radiopharmaceuticals for imaging), definition of the endpoint, range of absorbed doses and number of patients, could all be factors responsible for hiding correlations. Furthermore, the absorbed dose might not be the ideal quantity to consider. The transition from the absorbed dose as main parameter to start with to radiobiological quantities and models able to take into account DNA damage and repair mechanisms, the number and frequency of treatment cycles and other radiobiological effects, is thus a further challenge [110]. 

Multicentric trials are mandatory to explore the absorbed dose-effect correlation, overcoming the problem of the small number of patients [79]. Trials, in turns, require standardization of the dosimetry calculation methodology, together with the outcome/toxicity definition and data acquisition. 

Although at present each software package offers its own strategy for calculating the absorbed dose without well-established rationales or accuracy parameters to comply with, the whole scientific community—software manufacturers included—is making a great effort to disseminate recommendations and education materials with the shared goals of standardizing the methodology, identifying and, whenever possible, reducing the sources of error related to the absorbed dose calculation and improving the traceability of the dosimetric data [105,111,112,113]. From this perspective, the EANM provided several guidelines for dosimetry reporting, uncertainty analysis and dosimetric methods specific for some TRT therapies [51,62,77,114]. Other resources are in preparation. 

Despite difficulties, many different dosimetry-guided treatment planning protocols have been already developed for various therapies. Threshold doses are usually extrapolated from EBRT experience and possibly adapted to TRT in order to consider the different characteristics of the radiation delivery in the two treatment modalities (e.g., high dose rate vs. low dose rate) [9,115]. Furthermore, since TRT is often used for the treatment of metastatic diseases that present many lesions with highly different uptake and retention properties, treatments are usually planned based on OARs’ dose constraints more than lesions’ [7]. A possible approach for overcoming this problem could be to consider a “whole-body” tumor absorbed dose instead of the index lesion absorbed doses, as proposed by Violet et al. [48].

In addition, for a few therapies, the first promising signs are emerging, showing that the implementation of treatment planning based on dosimetry brings great benefits for the patients compared to the standard approach [43,116,117]. Garske-Román et al., e.g., proved that patients in which kidney-absorbed dose reached 23 Gy had a longer overall survival (54 vs. 25 months) and progression-free survival (33 vs. 15 months) compared to patients treated according to the standard protocol. For other therapies, instead, randomized controlled trials demonstrating the advantages of dosimetry-based versus fixed-activity approaches have not yet been performed. 

In conclusion, although proper dosimetry-guided treatment planning could not be applied yet in most therapies due to the lack of dose constraints or to challenging dosimetry, the increasing evidence of absorbed radiation dose-effect relationships and the recent successes of a dosimetry-based administration approach appear to be sufficient reasons to stimulate further research on the development of personalized treatment planning. 

In addition to dosimetry for treatment planning, post-treatment dosimetry is also to be considered as part of a personalized treatment. Since retreatment is frequently an option, the absorbed dose already delivered to OARs should be verified for each patient, according to the European Council Directive 2013/59/EURATOM. Moreover, although lesions and bone marrow dosimetry together with dosimetry for alpha-targeted therapy are still challenging [118], the increasing availability of new radiopharmaceuticals, technological improvement of imaging equipment and software for patient-based dosimetry makes patient-specific dosimetry for verification increasingly more feasible. 

Overall, the practice of internal dosimetry perfectly fits with the aim of precision and personalization, which are the major claims of medicine in the present era [109].

## 8. Conclusions

This paper provides an examination of methods and techniques used in TRT, with a focus on personalized dosimetry. Conceptual and practical tools for those who approach the field of internal dosimetry are presented, along with references of interest, especially regarding absorbed-dose–biological-effect studies. In order to fulfill the optimization principle and the recent European Council Directive 2013/59/EURATOM, personalized dosimetry in TRT should be integrated in clinical practice, as occurs in EBRT. Standardization of the dosimetric methodology, of the outcome/toxicity definition and of the data collecting procedure is required, in order to enrich the patient clinical data and make accurate multicentric studies feasible. These studies are fundamental to establish potential absorbed dose constraints for organs at risk and target tissues and, therefore, to improve patient outcomes and reduce long term costs. 

## Figures and Tables

**Table 1:** Summary of the main radiopharmaceuticals (RP) used in TRT, their therapeutic application and the corresponding administered activity in the treatment. Radiopharmaceuticals used for dosimetry are also reported. * Relating to a research protocol.

Therapeutic Application	RP for Therapy	Activity Typically Administered	RP for Dosimetry
Thyroid cancer	^131^I	1.1 to 5.5 GBq (thyroid remnant ablation) 5.5–11 GBq (treatment of metastases) single administration [23]	^123^I, ^124^I, ^131^I
Neuroblastoma	(^131^I)mIBG	3.7–11.2 GBq per cycle; multiple cycles [24]	(^131^I)mIBG
PRRT for NET and other somatostatin receptor expressing tumors	^90^Y-DOTATOC *	2.8–3.7 GBq per cycle; 4 cycles [25]	^111^In-DOTATOC
	^177^Lu-DOTATATE	7.4 GBq per cycle; 4 cycles [6]	^177^Lu-DOTATATE
Radioembolization of primary and secondary liver tumors	^90^Y resin or glass microspheres	2–4 GBq (resin) 3–20 GBq (glass) single administration [26]	^99m^Tc-MAA, ^90^Y microspheres
	^166^Ho poly-L-lactic acid microspheres	3.8 GBq/kg (liver weight) single administration [27]	^166^Ho microspheres
Radioimmunotherapy for hematologic malignancies (leukemia, MDS, myeloma, lymphoma)	^90^Y-MoAbs (Zevalin^®^)	11–14 MBq/kg (body weight) single administration [28]	^111^In-MoAbs
	^131^I-MoAbs (Bexxar^®^)	2.2–5.7 GBq single administration [29]	^131^I-MoAbs
Prostate cancer	^177^Lu-PSMA	3.7–9.3 GBq; 2 to six cycles [30]	^177^Lu-PSMA
	^225^Ac-PSMA *	4–13.4 MBq per cycle; 2–6 cycles [31]	imaging not possible, extrapolation from ^177^Lu-PSMA data
Bone metastases from breast and prostate cancers	^223^Ra	55 kBq/kg (body weight) per cycle; 6 cycles [32]	^223^Ra

**Table 2:** Examples of the variability in the absorbed doses following different TRT treatments reported by some authors.

Therapy	No. of Patients	Administered Activity	Absorbed Dose Range	Reference
^131^I for thyroid cancer	16	7.4 GBq	Tumor: 1–368 Gy	[34]
(^131^I)mIBG	53	666 MBq/kg	Red marrow: 2–5 Gy	[35]
^177^Lu-DOTATATE	777	7.4 GBq	Kidneys: 1–10 Gy	[6]
^177^Lu-DOTATATE	41	7.4 GBq	Tumor: 2–77 Gy	[36]
^90^Y resin or glass microspheres	40	0.4–2.4 GBq (according to empiric or BSA method)	Tumor: 40–495 Gy Healthy Liver: 1–100 Gy	[37]
^90^Y-MoAbs (Zevalin^®^)	72	15 MBq/kg up to a maximum of 1.2 GBq	Red marrow: 0.1–2.0 mGy/MBq	[38]
^177^Lu-PSMA	30	3.6–8.7 GBq per cycle	Parotid glands: 0.3–10 mGy/MBq Lesions: 0.03–78 mGy/MBq	[39]
^223^Ra (Xofigo^®^)	6	100 kBq/kg per cycle	Red Marrow: 177–994 mGy/MBq (from bone surface) 1–5 mGy/MBq (from blood)	[40]

**Table 3:** Most relevant examples of dose-effect correlations for different TRT cancer treatments reported in the literature.

Therapy	No. of Patients	Clinical Endpoint	Correlation Found	Reference
(^131^I)mIBG	26	Hematological toxiticy (Neutropenia)	Whole-body absorbed dose	[41]
^177^Lu-DOTATATE	14	Hematological toxicity (PLT ^1^ and WBC ^2^ variation)	Cumulative bone marrow absorbed dose	[42]
^177^Lu-DOTATATE	52	Hematological toxicity (PLT variation)	Per-cycle bone marrow absorbed dose	[43]
^177^Lu-DOTATATE	24	Tumor response (RECIST ^3^ criteria)	Tumor absorbed dose	[44]
^177^Lu-DOTATATE	48	Tumor response (CT)	Tumor absorbed dose	[45]
^90^Y microspheres	36	Tumor response (EASL ^4^ criteria) PFS OS	Tumor absorbed dose	[46]
^90^Y microspheres	24	Tumor response (^18^F-FDG PET/CT)	Tumor absorbed dose	[47]
^177^Lu-PSMA (mCRPC)	30	Tumor response (PSA, ^68^Ga-PET/CT)	“Whole-body” tumor absorbed dose	[48]
^153^Sm-EDTMP (bone metastases)	27	Hematological toxicity (CTCAE ^5^, PLT and WBC variation)	Bone marrow absorbed dose rescaled in terms of patient-specific trabecular volume	[49]
^223^Ra-Cl2 (mCRPC)	14	Tumor response (^99m^Tc-HDP)	Tumor absorbed dose in the first cycle	[50]

^1^ platelet; ^2^ white blood cell; ^3^ response evaluation criteria in solid tumors; ^4^ European Association for the Study of the Liver; ^5^ Common Terminology Criteria for Adverse Events.

**Table 4:** Software packages for phantom-based dosimetry.

Name	Availability	Decay Data	Number of Radionuclides	Phantoms	Specific Organ Models
OLINDA /EXM 1	Distributed by Vanderbilt University, presently withdrawn from the market	RADAR website [95]	Over 800	Cristy and Eckerman [100] + pregnant female series [101]	Peritoneal cavity, prostate gland, head and brain, kidney and spheres
Organ Dosimetry^TM^ with Olinda/EXM^®^ 2.0	Distributed by Hermes Medical	RADAR website [95]	Over 1000	RADAR phantoms [91] + animal phantoms	Peritoneal cavity, prostate gland, head and brain, kidney and spheres
IDAC 2.1	Free	ICRP 107 [94]	1252	ICRP 110 [92]	Spheres
3D-RD-S	Distributed by Rapid, LLC	ICRP 107 [94]	1252	ICRP 110 [92] and ICRP 143 [93]	Spheres
MIRDcalc	Free	ICRP 107 [94]	333	ICRP 110 [92], ICRP 143 [93] and weight-based phantoms	Spheres

**Table 5:** Main commercial software packages for patient-specific dosimetry.

Name	Manufacturer	Dose Conversion Method	Supported Therapy Radionuclides	CE/FDA Approval
SurePlan™ MRT	MIM Software Inc.	VSV	^177^Lu, ^131^I ^1^	CE/FDA
Planet^®^ Dose	DOSIsoft	VSV/LDM	^177^Lu, ^131^I	CE/FDA
Voxel DosimetryTM	Hermes Medical solutions	Semi-MC	^68^Ga, ^123^I, ^131^I, ^111^In, ^177^Lu, ^99m^Tc, ^90^Y, ^89^Zr, ^223^Ra, ^166^Ho	CE/FDA
QDOSE^®^	ABX-CRO	VSV	^11^C, ^15^O, ^18^F, ^44^Sc, ^64^Cu, ^68^Ga, ^86^Y, ^89^Zr, ^90^Y, ^124^I, ^89^Sr, ^99m^Tc, ^111^In, ^131^I, ^153^Sm, ^166^Ho, ^177^Lu, ^186^Re, ^188^Re	CE
SurePlan™ LiverY90	MIM Software Inc.	VSV/LDM	^90^Y microspheres ^1^	CE/FDA
Planet^®^ Dose	DOSIsoft	VSV/LDM	^90^Y microspheres	CE/FDA
Hybrid3D^TM^ SIRT	Hermes Medical solutions	LDM	^90^Y microspheres	CE/FDA
Simplicit90YTM v2.4	Mirada Medical	LDM	^90^Y microspheres	CE/FDA
VelocityTM Varian RapidSphere v4.1	Varian	DPK/LDM	^90^Y microspheres	CE/FDA
Q-Suite v2.0	QUIREM Medical BV	LDM	^166^Ho microspheres	CE

^1^ SurePlan™ allows uploading VSV kernels for other beta radionuclides for clinical or research purposes (e.g., ^90^Y, ^186^Re, ^188^Re, ^124^I for MRT and ^166^Ho for SIRT applications).

## Data Availability

Not applicable.
